# Continual updating and monitoring of clinical prediction models: time for dynamic prediction systems?

**DOI:** 10.1186/s41512-020-00090-3

**Published:** 2021-01-11

**Authors:** David A. Jenkins, Glen P. Martin, Matthew Sperrin, Richard D. Riley, Thomas P. A. Debray, Gary S. Collins, Niels Peek

**Affiliations:** 1 Division of Informatics, Imaging and Data Science, Faculty of Biology, Medicine and Health, The University of Manchester, Manchester Academic Health Science Centre, Manchester, UK; 2 NIHR Greater Manchester Patient Safety Translational Research Centre, The University of Manchester, Manchester, UK; 3 Centre for Prognosis Research, School of Primary, Community and Social Care, Keele University, Staffordshire, UK; 4 Julius Center for Health Sciences and Primary Care, University Medical Center Utrecht, Utrecht University, Utrecht, The Netherlands; 5 Centre for Statistics in Medicine, Nuffield Department of Orthopaedics, Rheumatology and Musculoskeletal Sciences, University of Oxford, Oxford, UK; 6 NIHR Manchester Biomedical Research Centre, The University of Manchester, Manchester Academic Health Science Centre, Manchester, UK

**Keywords:** Clinical prediction models, Dynamic model, Validation, Model updating, Model development, Learning health system

## Abstract

Clinical prediction models (CPMs) have become fundamental for risk stratification across healthcare. The CPM pipeline (development, validation, deployment, and impact assessment) is commonly viewed as a one-time activity, with model updating rarely considered and done in a somewhat ad hoc manner. This fails to address the fact that the performance of a CPM worsens over time as natural changes in populations and care pathways occur. CPMs need constant surveillance to maintain adequate predictive performance. Rather than reactively updating a developed CPM once evidence of deteriorated performance accumulates, it is possible to proactively adapt CPMs whenever new data becomes available. Approaches for validation then need to be changed accordingly, making validation a continuous rather than a discrete effort. As such, “living” (dynamic) CPMs represent a paradigm shift, where the analytical methods dynamically generate updated versions of a model through time; one then needs to validate the system rather than each subsequent model revision.

## Background

Clinical prediction models (CPMs) are tools that compute the risk of an outcome given a set of patient characteristics (“predictors”), and can be used for informing diagnosis or prognosis in individuals [1, 2]. They are typically based on multivariable regression models, for example as derived by analysing historical cohort data or routinely collected healthcare data. Arising from the desire to move health systems away from managing or curing disease towards preventative medicine, CPMs have become popular and several are now embedded in clinical practice (e.g. QRISK3 [3] and the Leicester diabetes risk score [4]).

Commonly, the process of developing a CPM equation is a one-time activity, with estimates of model parameters obtained from a single dataset ignoring time. Once a model has been developed, usually the model equation remains fixed until a revision is conducted. However, revisions are rare and usually undertaken at an arbitrary time, or following an external validation that suggests the model is miscalibrated. Model validation is an important aspect of the CPM pipeline and aims to evaluate whether model predictions are accurate (in settings they would be applied to in practice). Similarly to model development, validation is often a one-time activity. Commonly, the literature refers to CPMs as being “validated”, but this may create a false impression that no more model testing needs to be performed. In this paper, we propose moving away from one-time model development and validation, and rather embed CPM development, validation, and updating into a dynamic system that reflects an evolving healthcare service. For example, the current COVID-19 pandemic represents a situation where this would be particularly useful, given how quickly healthcare processes have changed, meaning that any prediction models for COVID-19 need to be updated rapidly [5, 6]. For example, in the future, vaccinations, immunity build up, and virus mutation may affect the strength of predictor effects over time.

## Calibration drift prediction problem

CPM production pipelines are built on the assumption that once produced and verified, evidence can be translated into practice ad infinitum. But the distribution of patient characteristics, disease prevalence, and health policies change over time. When these changes occur, the estimated CPM parameters and corresponding predictions may no longer be valid [7, 8]. Consequently, the agreement between the observed and predicted event rates worsens over time [9]: the so-called calibration drift [10]. Hickey et al. [8] highlight this issue in the logistic EuroSCORE model [11], which quickly became outdated as improvements in patient outcomes were rapid. Therefore, there is evidence that model coefficients need to change through time, as illustrated with EuroSCORE. In addition, Luijken et al. [12] observed that changing predictor measurement procedures induced miscalibration in nine real-world examples.

Traditional practice to address this is to develop another CPM de novo. However, alternative approaches, such as updating [13, 14], aggregating existing CPMs [15, 16], or meta-analysis of individual participant data [17, 18], are preferable because they do not discard historical data and previous research efforts [19]. For example, models such as QRISK are now updated yearly [3, 20] using contemporary data and also revised to include additional predictors (such as the revision of QRISK2 [21] into QRISK3 [3]). Nonetheless, this updating (recalibration) is still relatively uncommon, often occurs a substantial time after model development, is often undertaken at arbitrary time points, and is typically dependent on funding. For example, EuroSCORE II [22] was developed in 2012, some 13 years after the original model, and it is unclear when this will be updated again. The problem with this approach to model validation and revision is that predictive performance of a CPM may only be investigated many years after the model has been developed. Although this can subsequently result in the CPM being updated, incorrect decisions may have already been made as a result of the miscalibrated model and harm already caused.

Typically, a model is developed or updated under the assumption that the data are well described by a fixed underlying model where the coefficients are constant across the observation period used to develop the model. If the prevalence of an outcome is increasing at a steady rate during a 5-year window of data collection and then used to develop the model, the CPM will be calibrated to the middle of the window and not the most recent data. The overarching issue here, for both development and validation, is that the data-generating process could change through time. While frequent model updating will mitigate these issues, it does not eliminate the problem since commonly used methods do not acknowledge temporal changes. Rather, we propose embedding prediction models in practice to ensure development, validation, and updating are a continual process. We now discuss how this might be implemented and the challenges involved.

## Possible solution and challenges

The healthcare system and disease populations are constantly changing, but the CPMs we deploy are not updating at the same rate. Therefore, we need to ensure a CPM is maintained on a continual (rather than an ad hoc) basis. For this to be achieved, we need to reduce the latency period between observing calibration drift and updating a model, thus moving towards a service that constantly monitors a model and has an embedded feedback loop where the monitoring information is then relayed back to the model and used to modify and maintain it.

### Dynamic models

Dynamic prediction models have been proposed as a potential solution to calibration drift and to allow prediction models to evolve simultaneously with the healthcare system [23, 24]. They are a collection of analytical methods that allow CPMs to continuously adapt as data on new patients arises—thus reducing the data-action latency compared with traditional methods of developing CPMs at a single point in time. By dynamic model, we mean models that update over calendar time as data on new individuals arises, not models that update predictions for individuals as new data on them arises. A dynamic model is formulated to account for the calendar time that a prediction is made, that is the calendar time predictors are recorded for each individual (e.g. date of GP appointment), and is designed to evolve over time, such that the parameter estimates are not constrained to remain fixed as (calendar) time evolves. Thus, given a fixed set of patient characteristics, a dynamic model could produce different predicted risks at different times of prediction, for example, if two individuals with the same predictor values are observed at different times, then the model could produce different predicted risks.

The simplest approach to develop a dynamic CPM is to include (calendar) time as a predictor [25, 26]. Alternatively, the Bayesian dynamic model could be implemented, where information obtained from past data is used as prior information and combined with new data to obtain updated estimates, thereby updating with new observations in real time [23, 24, 27]. More weight can also be given to the most recent data by “forgetting” past data at a given rate. For more detail on these methods, see the reviews by Jenkins et al. [24] and Su et al. [28]. In summary, dynamic models allow us (1) to utilise historical data and models more effectively, (2) to reduce data-action latency (time between changes in the data and reacting to them), and (3) to “automatically” adapt model parameters over time. Hickey et al. [29] illustrate the use of dynamic modelling in EuroSCORE and show how the coefficients change over time.

Although there is much potential in dynamic models, they are rarely used in healthcare. There are both methodological and practical reasons why this is so. Methodological reasons include the following: (1) a lack of methods on how to validate dynamic prediction models [24], (2) uncertainty on when to include new or exclude existing predictors, (3) deciding how much to discount historical data, (4) uncertainty around when to update the model, (5) the potential lack of model transparency, and (6) inconsistent outputs over time (e.g. a patient with the stable risk factors could have changing predicted risks because the model has changed). Practical considerations include the following: (1) lack of robust and suitable new data to be able to update the models continuously, (2) complexity of the dynamic modelling approach, (3) lack of software implementations, (4) lack of requisite expertise by those developing the model, and (5) lack of infrastructure and funding. However, many of these problems are not specific to dynamic CPMs, for example, the problem of how to handle historical data in traditional CPMs is often ignored but a problem is still present. When updating CPMs, we often append the new data to past data or use only the recent data to perform the update. This is an arbitrary choice by the researcher performing the update, and neither is likely to be optimal. Raftery et al. [23] attempted to address this in dynamic modelling by using an approach to choose how to discount past data at each update by optimising the predictive performance over past samples, but this is computationally expensive. More of these challenges have also attempted to be addressed in statistical literature, for example, use of the time dependent AUC [30], but have yet to be applied to continual prognostic modelling. Other theoretical methods to address these challenges also exist, but their application in prognostic model research is generally lacking and it remains unclear how this would and should affect prediction model research.

### Model surveillance

If a dynamic model evolves with every new data point, then there is only ever the next data point in which to validate each evolution of the model. Furthermore, validation at a given time point is only a single snapshot in time. It does not follow that if a CPM, dynamic or otherwise, has high performance at a given point in time that it will always perform well. However, as we continue to make predictions for new patients, we can record and monitor the accuracy, essentially continuously monitoring and testing for calibration drift (prequential testing [31]). This leads to the idea of model surveillance, where the CPM monitoring could be performed after every new data point or at given intervals. Prequential testing approaches have a long history in the statistical literature and have been used in areas such as economic forecasting. However, they have yet to be transported and used in prediction model research. Lenert et al. [32] discuss the notion of having surveillance of models used in practice as the models themselves can directly impact the data and subsequently their own performance. They explain that without surveillance, models will have limited effectiveness and can become hazardous. We propose prequential testing as a potential solution to these issues but further research is required.

### Feedback loop

**Fig. 1:**
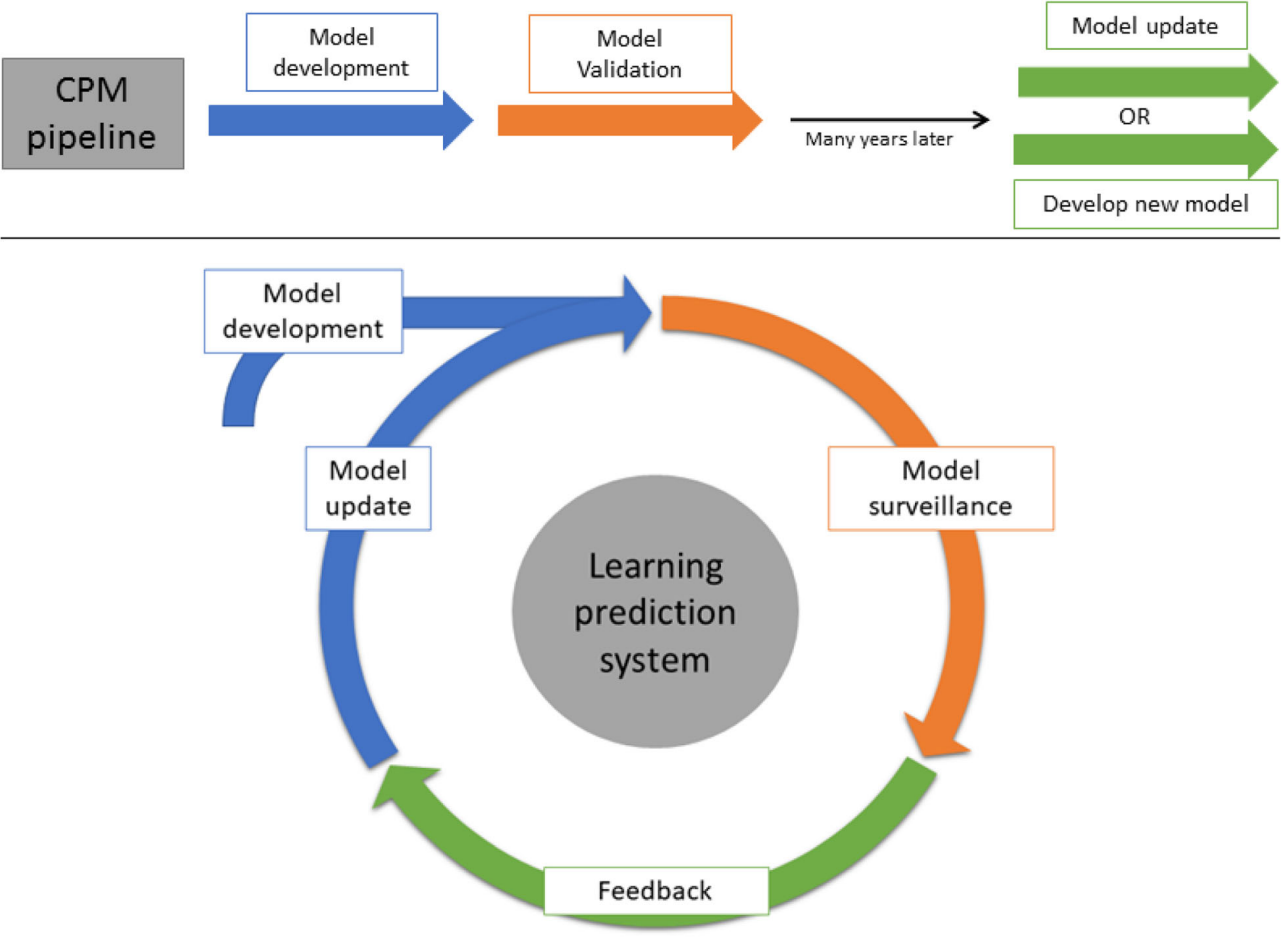
Illustration of the current CPM pipeline (top) and the proposed learning prediction system (bottom)

Model surveillance, and the use of prequential testing, could also allow us to address some of the issues discussed above. However, continuous monitoring of performance will not address all of these problems. The results of continuous monitoring need to be transported back into the model providing a feedback loop, which allows the model to learn and ensures the model continually provides accurate predictions (Fig. 1). Ideally, this would be conducted in a timely manner to reduce the data-action latency, which is a key metric of the learning health system (LHS) [33], a system that improves itself by learning from new data through cyclic processes that mobilise data to create new knowledge and then use that knowledge to improve. We therefore need a system approach, where one encompasses clinical prediction modelling into a learning health system, thus resulting in a learning prediction system. This system could improve itself by learning from data, continually and in real time, and would take place through cyclical processes (Fig. 1).

Minimising the data-action latency, and doing so efficiently, requires concerted data capture, aggregation, and analysis followed swiftly by interpretation of results, assignment of responsibility for any actions, and recording of actions. Not only can a learning prediction system allow a model to evolve over time, but it could also decide when and how to evolve each iteration of the cycle. This is achievable in LHSs that are supported by infrastructures that enable these processes to take place routinely and with efficiency of scale and scope. Dynamic methods (updating and/or monitoring) offer a flexible solution, requiring less manual labour, but need the infrastructure and sustained resources in place to implement them. Adibi et al. [34] discuss an integrated infrastructure for CPMs and highlight that much of the technology is available, but not yet fully utilised in healthcare. For dynamic updating to work, a system is needed where patient data is automatically collected and stored in a database and subsequently used to update parameter estimates.

### Further considerations

We acknowledge that continually updating a CPM might not always be needed. For example, comparative audit requires a standardised method to adjust for case-mix differences, so dynamic methods might not be appropriate. Also, updating all of the coefficients in a model may not always be a good idea. Booth et al. [35] recently proposed temporal recalibration in settings where survival is improving over time. This approach develops a model using all the available data but then recalibrates the baseline survival function using a subset of the data from a recent time window. Vergouwe et al. [36] described a closed test procedure to select methods for updating prediction models, something which could be embedded into the learning prediction system. This study also found that model revision, updating all model coefficients, can be chosen over intercept-only updating, even in small sample sizes, further supporting the need for a continual system. Although we could redevelop or update traditional models on a daily basis, the use of dynamic methods may offer a more flexible solution. Both traditional and dynamic approaches to CPM development/updating have their advantages and disadvantages (see Table 1), but ultimately, all CPMs need their performance to be monitored regularly and thus require a continual flow of data.

**Table 1:** Summary of the characteristics and pros and cons for different modelling approaches

Models	Characteristics	Advantages	Disadvantages
**Existing approaches**			
Fixed model never updated	• Model and coefficients fixed • Never updated	• Cheap (funding available) • Low complexity and easy to communicate	• Can become miscalibrated quickly • Dethroned by new model likely developed in future • Ends up as research waste • Loss of information
Model with ad hoc updating (e.g. EuroSCORE)	• Updated when opportunity allows • Fixed coefficients between updates	• Easy to maintain • Cheap (funding available) • Low complexity • Little manual labour • Advantageous over developing a completely new model	• Non-responsive to calibration drift • Long data-action latency
Models that get periodically updated (e.g. QRISK)	• Fixed regular updates • Set time period between updates	• Lower chance of miscalibration than above • Allows predictors to be included/excluded from the model • Relatively low complexity	• Funding required • Can still observe calibration drift between updates • Increased maintenance • Requires more than manual labour to maintain • Uncertainty on length of time needed between updates
**Proposed approaches**			
Models with discrete updating and continual validation/monitoring (learning prediction system with discrete updating and continual monitoring)	• Updated when opportunity allows • Continuously monitors new data • Updated as a result of the monitoring • Feeds back information to the model on how and when to update	• Monitoring informs updates • Only update when required • Reactive to changes • Transports well across settings and populations	• Funding and infrastructure required • Update does not immediately follow after suggestion from monitoring • Requires some manual labour to maintain
Complete dynamic system (continual model update with continual validation/monitoring) (learning prediction system with continual updating and monitoring)	• Dynamic model • Continuously monitors new data • Feeds back information to the model	• Efficient • Potential to be more accurate • Provides less miscalibrated results • “Reacts” quicker to change (responsive) • Possible to automate • Less manual labour to maintain • Transports well across settings and populations • Do not need to store the data	• Requires access to an appropriate “living” data source that is linked to the relevant outcomes • Uncertainty on how one should validate dynamic prediction models • Uncertainty on when to include/exclude predictors • Deciding how much to discount historical data • Uncertainty around when to update the model • Lack of software packages • Complexity of approach • Lack of requisite expertise by those developing the model • Lack of transparency • Inconsistent outputs from day to day • Funding

Dynamic CPMs require a continual flow of data. These are typically provided by routine data sources such as audit data, registries, and electronic health records. Dynamic CPMs also offer opportunity in remote monitoring data, such as wearable device or app data, which provides large quantities of data in real time that is otherwise challenging to analyse. However, continuous data flows are usually not supported by epidemiological studies and clinical trials. This could raise concerns about the quality of dynamic CPMs because routine data sources tend to have poorer data quality and higher levels of missingness than study datasets. A possible solution is to develop CPMs using high-quality study data (e.g. from a prospective observational study) and dynamically revise and monitor them using the routine data. However, quality checks and comparisons between the datasets would still be required.

Throughout this article, we have focused on the temporal aspect of miscalibration; however, miscalibration can also occur when CPMs are transferred to different settings and/or populations [9, 37]. It may be possible to generalise the concept of dynamic CPMs to address this type of calibration variation in space. For example, dynamic approaches could be used to tailor a model to a local population or transfer a model to a different setting. This is an area that requires further research.

## Conclusion

Static CPMs are at risk of being always one step behind on reality. Through an alliance between information technology and statistics, clinical prediction can be progressed to a continual service that minimises the data-action latency in preventative medicine.

## Data Availability

Not applicable
